# Spontaneous Quadriceps Tendon Rupture Associated With Severe Secondary Hyperparathyroidism in a Patient on Chronic Hemodialysis: A Case Report

**DOI:** 10.7759/cureus.112230

**Published:** 2026-07-07

**Authors:** Ismail Ait Elkihal, Nouhaila Charafi, Mehdi El Mansouri, Nabil Hamouche, Mariam Chettati, Wafaa Fadili, Inass Laouad

**Affiliations:** 1 Nephrology, Mohammed VI University Hospital Center, Faculty of Medicine and Pharmacy, Cadi Ayyad University, Marrakech, MAR

**Keywords:** chronic hemodialysis, ckd-mineral and bone disorder (ckd-mbd), end-stage chronic kidney disease, extensor tendon rupture, minimal trauma

## Abstract

Spontaneous or minimal-trauma ruptures of the quadriceps tendon are an exceptional clinical entity, representing a highly unusual presentation even among patients with underlying metabolic conditions, including uremic patients on chronic hemodialysis. We report the case of a 40-year-old male patient on chronic hemodialysis with severe secondary hyperparathyroidism, who was admitted for a rupture of the left quadriceps tendon following minimal trauma. Treatment consisted of the surgical reconstruction of the extensor apparatus and management of his hyperparathyroidism.

## Introduction

Rupture of the quadriceps tendon predominantly affects middle-aged men following significant trauma. However, an increased incidence of this condition is strongly associated with systemic comorbidities such as diabetes mellitus, gout, rheumatoid arthritis, systemic lupus erythematosus, and end-stage renal disease (ESRD) [1]. Although its precise incidence remains difficult to quantify due to its rarity, spontaneous tendon rupture is a distinct clinical entity in chronic hemodialysis patients, mostly documented through limited case series and literature reviews [1,2].

In ESRD, secondary hyperparathyroidism commonly develops as a result of chronic hypocalcemia, hyperphosphatemia, and diminished vitamin D activation. While the exact pathogenesis is still debated, elevated parathyroid hormone (PTH) levels play a central role [2]. Specifically, chronic hyperparathyroidism triggers subperiosteal bone resorption at tendinous insertion sites, structurally weakening the osteotendinous junction and predisposing patients to failure under minimal stress. Although the Achilles, patellar, and triceps tendons are also frequently affected in this metabolic milieu, quadriceps involvement causes severe functional loss [2,3]. This underlines the clear need for early prevention and optimal management of mineral and bone disorders to avert such debilitating musculoskeletal complications.

Here, we report the case of a chronic hemodialysis patient presenting with a complete rupture of the left quadriceps tendon following minimal trauma.

## Case presentation

A 40-year-old male patient with a history of ESRD of indeterminate etiology was admitted for the management of severe left knee pain accompanied by complete functional inability. He had been undergoing hemodialysis for eight years (two sessions per week, based on the patient's informed choice) and had a known history of secondary hyperparathyroidism. The injury occurred following minimal trauma, specifically a fall from standing height with a direct impact on the knee. Prior to this event, the patient was fully autonomous and ambulatory.

Clinical examination revealed a swollen left knee with a palpable, painful suprapatellar gap. The patient was completely unable to perform active extension of the left knee and could not lift his heel from the bed. There were no clinical signs of associated meniscal or ligamentous damage, no open wounds, and no neurovascular deficits.

Laboratory investigations showed a total serum calcium level of 2.58 mmol/L with total protein at 62 g/L, the serum albumin level was low at 32 g/L, yielding a corrected serum calcium level of 2.78 mmol/L. Other notable laboratory values included significantly elevated phosphorus at 2.74 mmol/L, an exceptionally high iPTH level at 2344.70 pg/mL, and serum ferritin at 51.86 ng/mL. The complete blood count revealed a hemoglobin level of 10 g/dL, characterized as a normochromic, normocytic, and non-regenerative anemia, typical of chronic kidney disease anemia. Viral serologies for hepatitis B virus (HBV), hepatitis C virus (HCV), and human immunodeficiency virus (HIV) were all negative.

In response to these findings, oral calcium supplementation and active vitamin D therapy were discontinued. The patient was subsequently started on calcimimetics along with a non-calcium-based phosphate binder (sevelamer), and a low-calcium dialysate was initiated. Concurrently, management of chronic kidney disease anemia was optimized, and the patient was maintained on erythropoiesis-stimulating agents (ESA), and intravenous (IV) iron supplementation was administered to correct the iron deficiency. However, medical therapy failed to achieve effective control of the severe secondary hyperparathyroidism. Due to this persistent refractory hyperparathyroidism, a parathyroidectomy was performed, which successfully led to the improvement of the patient's bone and mineral metabolism disorders.

**Figure 1:**
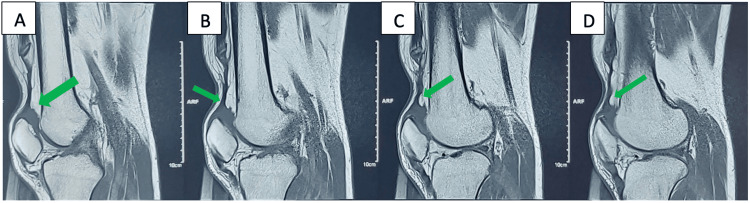
Magnetic resonance imaging (MRI) of the left knee. (A, B, C, D) Sagittal views demonstrating a complete rupture of the quadriceps tendon near its superior patellar insertion, resulting in a significant proximal tendon retraction of 5 cm. The tendon rupture and its proximal retraction, along with the avulsed bone fragment from the superior pole of the patella (clearly identified in panels C and D), are indicated by the green arrows.

A standard lateral X-ray of the left knee demonstrated patella baja with no associated fractures. Magnetic resonance imaging (MRI), performed to confirm the diagnosis and evaluate the precise location of the tear, revealed a complete rupture of the quadriceps tendon with bone avulsion at the superior pole of the patella, resulting in a proximal tendon retraction of 5 cm (Figure 1).

Following a thorough preoperative assessment and after obtaining informed consent, the patient was taken to the operating theater. Under spinal anesthesia and with a pneumatic tourniquet applied at the root of the thigh, an anterior midline vertical surgical approach was performed. The surgical procedure consisted of an end-to-end suture utilizing "U" stitches in opposite directions with No. 2 Vicryl suture, reinforced by a continuous locking suture using the same material. The wound was closed over a suction drain.

The postoperative course was uneventful, with no thromboembolic or infectious complications. The operated limb was immobilized in a splint with the knee in full extension for six weeks. Passive rehabilitation was initiated early, while active rehabilitation commenced after the six-week immobilization period.

At the three-month postoperative follow-up, recovery was still partial; active rehabilitation progressed slowly and was relatively delayed due to the patient's frail underlying condition. At this stage, the patient achieved limited independent ambulation with a walker, and the knee range of motion (ROM) showed a persistent extension lag of approximately 15 degrees, with passive flexion restricted to 60 degrees.

By the six-month postoperative evaluation, a favorable clinical condition was observed. The patient demonstrated significant improvement in motor function and lower limb strength, achieving complete, unassisted ambulation with a stable gait. The knee joint showed a satisfactory range of motion, achieving full active extension (0 degrees) and active flexion up to 100 degrees.

## Discussion

The exact pathogenesis of tendon ruptures in hemodialysis patients has not yet been fully elucidated, but several pathophysiological hypotheses have been proposed. These include degenerative tendon remodeling linked to chronic acidosis [4] and erosive enthesopathy related to secondary hyperparathyroidism. Furthermore, ruptures in uremic patients typically occur at an earlier age compared to non-uremic populations [5,6].

Preston and Adicoff suspected calcium deposits to be the origin of tendon tissue weakening [7]. Finlayson et al. studied the effects of chronic acidosis in chronic renal failure on connective tissue and found it to be associated with connective tissue elastosis [4].

Secondary hyperparathyroidism is strongly implicated in tendon weakening. Malta et al. highlighted that hemodialysis patients with quadriceps tendon ruptures exhibit significantly higher serum levels of iPTH and alkaline phosphatase compared to matched controls. This severe hyperparathyroidism leads to sub-tendinous bone resorption, which critically weakens the tendon insertion site at the bone-tendon junction [8]. This mechanism is consistent with our patient's presentation, given his exceptionally elevated iPTH level (2344.70 pg/mL) and the bone avulsion at the superior pole of the patella.

Recent literature also underscores the crucial role of chronic inflammation in tendon vulnerability [9,10]. Malta et al. found that patients with tendon ruptures had a higher prevalence of chronic Hepatitis C infection, along with significantly lower serum levels of albumin and hemoglobin - key markers of chronic inflammation [8]. Similarly, Basic-Jukic et al. reported that a large proportion of their patients who suffered from spontaneous tendon ruptures were also chronic carriers of the Hepatitis C virus [9].

Moreover, pharmacological treatments frequently prescribed to this population can severely amplify the risk of rupture. Basic-Jukic et al. demonstrated that the use of systemic corticosteroids and fluoroquinolone antibiotics (such as ciprofloxacin) are major additive risk factors that predispose uremic patients to tendon injuries [9]. Fluoroquinolones, in particular, should be avoided in patients with existing hyperparathyroidism. Additionally, statin therapy, commonly used for dyslipidemia, has occasionally been linked to tendinopathies and may further contribute to tendon weakness [10].

From a therapeutic standpoint, reconstructive surgery is the treatment of choice to restore knee function. Early surgical reconstruction combined with appropriate rehabilitation maximizes functional recovery. However, even in cases of delayed surgical intervention, patients can achieve satisfactory functional outcomes and regain independent walking ability without crutches [11].

Ultimately, strict medical or surgical management of secondary hyperparathyroidism remains essential to prevent these severe complications.

## Conclusions

The combined effects of end-stage renal disease and severe secondary hyperparathyroidism may result in significant tendon weakening, predisposing patients to spontaneous or minimal-trauma ruptures. This condition involves a multifactorial pathogenesis, including chronic acidosis, prolonged dialysis, secondary hyperparathyroidism, and chronic inflammation. Early surgical repair followed by tailored rehabilitation is essential to achieve satisfactory functional recovery. To prevent this debilitating complication, optimal medical and surgical management of secondary hyperparathyroidism is of paramount importance.
